# Re-Education in Walking

**Published:** 1917-02-10

**Authors:** James B. Mennell


### RE-EDUCATION IN WALKING.

By JAMES B. MENNELL, M.A., M.D., B.C.Cantab., etc.

In pre-war days there existed a tendency on the part of the surgeons of this country to relegate all the later part of the treatment of injuries to a massage-worker, and to interest themselves no more in the patient's career once the immediate surgical treatment had ceased. Thus few appreciated how tedious and slow was the recovery of patients, even after the receipt of comparatively trivial injuries.

With the advent of a host of wounded in the country, the medical profession was faced with a problem which was, therefore, a comparatively new one to many of its members. The patients could no longer be discharged from hospital semi-cured, or sent to the physical exercise department of the hospital, or put under the care of a masseuse with an almost instinctive hope that they would not be heard of further. Treatment had to continue till the patient was fit once more to return to military duty, and, amongst other things, until he no longer limped. It has come as a very unwelcome surprise to many how long and difficult is the task of eradicating a limp once it has been acquired.

### The Wasting of Muscles.

We must, I think, take it for granted that after any injury (no matter how trifling provided that the effect can be transmitted through the superficial structures) wasting will take place to a greater or less degree in the underlying muscles. Moreover, if a joint is injured, wasting will take place in the muscles that control the movements of that joint. This is largely reflex, as the nerves that supply the joint are the same as those that supply the muscles controlling its movement. Some muscles are always more affected than others; as a general working law, we may take it that the muscles containing the coarser fibres will suffer the most.

Not only is a wasted muscle more feeble than its fellows, but it also exhibits a longer latent period between the receipt of stimulus and its contraction. Hence, if a leg has been injured, many points have to receive attention if we desire to restore co-ordination and thus prevent the patient from limping.

First and foremost, of course, we must try to train up the strength of the weakened muscles. But in the meantime much can be done to restore co-ordination by training the less affected muscles to adapt the strength of their contraction to that of their weaker fellows. The latter have to be taught to respond more rapidly to the receipt of stimulus; while the former have to learn, as it were, to await the effect of the arrival of the stimulus in their more sluggish brethren. When movement is painful, another element is introduced. Now the muscles may respond strongly and with usual rapidity to their stimulus; but when a certain point is reached pain is felt, and reflexly the contracting muscles lose their contraction and relax suddenly and unexpectedly. The patient says that the joint feels weak.

A patient with a hallux rigidus, for example, feels pain whenever the first metatarso-phalangeal joint is bent. Therefore he takes every care instinctively to avoid this occurrence, and walks either with the inner border of the foot raised or with the foot everted. In the former case the invertors are held in spasm as also are the flexors of the toes, and there is undue rigidity of the whole limb. In the latter instance the knees are kept somewhat bent, and the gait is characterised by a general slouch. Some attempt is made at true heel and toe walking, the "toe" element being reduced to a minimum, and concerning only the outer toes. If the knee is kept stiff, the first trace of pain in the toe causes the knee to relax and it bends suddenly forward.

In double flat-foot, uncomplicated by hallux rigidus, the knees are kept bent, and all attempt at "heel and toe" work is abandoned, the patient planking the foot down flat at each step. The feet are kept fully everted. In the gait of the one-flat-foot man one leg works normally and the other as before.

Walking with foot-drop is again a thing apart, and calls for an undue raising of the knee with each step. Even when the dorsi-flexors of the toes have recovered a certain amount of their lost tone, this gait is maintained.

Functional spasm of the invertors is usually associated with a considerable amount of equinus, and also entails a slight degree of outward swinging of the whole limb. If the ankle is stiff, one of two things may happen. Either the patient will walk with a stiff knee and the foot fully everted, or with the knee bent and the foot only partly everted. The most natural walk with a stiff knee is to swing the foot outwards in a semicircle, but the patient usually adopts, unless taught to do otherwise, the more pronounced limp due to rising on the toes of the sound limb to an undue extent.

### Weak Knee and Weak Quadriceps.

The weak knee is usually synonymous with weak quadriceps. All goes well with the step till the weight is transferred from the back leg to the forward. Then the knee, which should remain rigid, suddenly bows forward. This constant and unnatural bending at a most inauspicious moment is bad enough; but, when we remember that the vastus internus is almost invariably wasted out of all proportion to the rest of the quadriceps, and that thereby the direction of the movement of the patella is rendered out of truth, it is small wonder that these patients suffer from recurring synovitis at frequent intervals. As a pure aside I may mention that there is a somewhat widespread belief that the vastus internus cannot be efficiently exercised as a separate entity. It can be done in three ways. First, during all attempts to bend the knee backwards when once the limit of movement in this direction is reached. Second, by tiptoe walking, the knees being kept stiff. Third, in the free standing position by slightly flexing the knee and then circling it inwards.

The limp of the dislocated hip is probably familiar to us all. Nothing can avert a marked limp, but much can be done by education to keep it within reasonable limits.

When the hip-joint is fixed the patient has to learn to walk by tilting and swinging the pelvis. In the early stages, the whole limb is kept rather rigid, and the limp is not very pronounced. As time progresses, unless the danger is pointed out, the whole of the rest of the body is allowed to fall loose and to partake in the general swing. The limp is then very accentuated, and has every appearance of being that of a man with a shortened leg.

Time does not admit of demonstrating the full details of re-education applicable to each individual type of injury that is calculated to lead to any one form of limp. I propose, therefore, to imagine that one limb only has become generally wasted, while the other is unaffected. Our task is to train up the muscles that will inevitably be more wasted than their fellows to the standard of the latter, and to train down the more favoured muscles to meet the needs of their less favoured fellows. This entails the double consideration of strength and the duration of latent period. It corresponds to training a company on the march to accommodate itself to the activities of its weakest member. Meantime the sound limb must be educated down to meet the needs of the weakened limb, while the latter is trained up as a whole to the level of its fellow.

### Use of Remedial Apparatus.

The first thing to be done is to teach the patient to swing his legs, first alternately and then together. It is a difficult art to teach, and illustrates at once how great is the loss of co-ordination. The two exercises are then combined and ankle movement is added. The patient is then taught to interrupt the exercises at the word of command and to maintain the position in which a halt is called while slow counting proceeds. He is next allowed to exercise on the sliding-seat. At first the rails are placed almost parallel to the floor, and the elevation is increased day by day. The foot-piece is kept loose. As soon as the full elevation has been attained it is once more lowered, and the foot-piece is fixed at an angle suited to the requirements of the case. The elevation is once more gradually increased.

After this, or perhaps meanwhile, the patient is allowed to sit on a chair with the legs crossed while the feet rest with their outer borders on the ground. He is then instructed to claw with the toes. It sometimes helps a patient to understand what is meant if he is told to try to imitate a monkey trying to climb up a rope. The feet are then placed on the floor in front of the patient, care being taken to see that most of the weight rests on the outer side of the foot, or rather that no weight is allowed to press downward the longitudinal arch. The heels remain on the ground while the toes are raised alternately and then together. Later the movement is combined. The feet are then drawn backwards by slow stages, the toes being kept turned inwards, and the heels are then raised and lowered in similar manner.

The next stage presents great difficulty unless co-ordination is well established. It consists of combining the previous exercises, so that the patient executes a species of clog-dance. A return to the clawing movement is now made, and the patient raises himself by the arms for a few inches from the seat of the chair. Gradually he rises higher and higher till finally the full weight rests upon his feet for the first time. Now he may be allowed to practise the taking of a full walking step. The only preliminary left is to swinging of the whole leg. At first he will keep knee and ankle stiff. He must be taught to bend the knee with each movement of the hip, and then to bend the ankle in co-operation with the two larger joints.

This accomplished, the first quarter of a step must be practised. It consists of a continuance of the swinging, but every now and then at first, and at every swing later, the heel of the foot is brought to a full stop by being brought into contact with the floor. All these exercises are best performed when the patient uses a board as a balancing-pole.

The next quarter-step is added, as soon as proficiency has been attained in the performance of the first quarter, by repeating this and then by lowering the toe of the advanced foot while raising the heel of the one behind. The patient is then taught to rock to and fro, the foot of the injured limb being used as the forward foot at first, and its stronger fellow taking this position later. The tendency to rise on the toes of both feet as the body swings forward must be strenuously combated.

Patients are usually rather slow getting over this stage, so fully is co-ordination required for its efficient performance. The last two quarters are soon learnt. The first two are repeated, and then, instead of rocking back, the rearmost foot is swung forward and the heel placed in contact with the ground. The rocking then takes place throughout the three-quarters of the step that have thus been accomplished. Very soon the step can be completed and again the full "rock" to and fro is performed. All this time a support has been used, a chair that runs smoothly on the floor being all that is really required, though the apparatus shown is a valuable asset.

### Patients Take Speedily to Exercises.

I fear all this has sounded very complicated. It is not really so. The whole art can be learnt quite quickly by any intelligent masseuse in a few days, and it has surprised me not a little to find how speedily patients take to the exercises. It must be that there is something in human nature that prompts them to overcome their deficiencies, or that resents being beaten by such trivial feats as they are called upon to perform. One great secret of the success of the plan is doubtless the careful graduation of the increase of movement. I feel sure, moreover, that were some such system adopted as routine in the after-treatment of all those who have sustained any injury to the lower limb, that recovery would be more rapid, and that limping would be less noticeable amongst our patients.

